# Optogenetic dissection of medial prefrontal cortex circuitry

**DOI:** 10.3389/fnsys.2014.00230

**Published:** 2014-12-09

**Authors:** Danai Riga, Mariana R. Matos, Annet Glas, August B. Smit, Sabine Spijker, Michel C. Van den Oever

**Affiliations:** Department of Molecular and Cellular Neurobiology, Center for Neurogenomics and Cognitive Research, Neuroscience Campus Amsterdam, Vrije University AmsterdamAmsterdam, Netherlands

**Keywords:** optogenetics, prefrontal cortex, cognition, depression, addiction, fear, memory

## Abstract

The medial prefrontal cortex (mPFC) is critically involved in numerous cognitive functions, including attention, inhibitory control, habit formation, working memory and long-term memory. Moreover, through its dense interconnectivity with subcortical regions (e.g., thalamus, striatum, amygdala and hippocampus), the mPFC is thought to exert top-down executive control over the processing of aversive and appetitive stimuli. Because the mPFC has been implicated in the processing of a wide range of cognitive and emotional stimuli, it is thought to function as a central hub in the brain circuitry mediating symptoms of psychiatric disorders. New optogenetics technology enables anatomical and functional dissection of mPFC circuitry with unprecedented spatial and temporal resolution. This provides important novel insights in the contribution of specific neuronal subpopulations and their connectivity to mPFC function in health and disease states. In this review, we present the current knowledge obtained with optogenetic methods concerning mPFC function and dysfunction and integrate this with findings from traditional intervention approaches used to investigate the mPFC circuitry in animal models of cognitive processing and psychiatric disorders.

## Introduction

Detailed insight into the connectivity and functionality of the nervous system is of pivotal importance for understanding how the brain functions in health and disease states. The medial prefrontal cortex (mPFC) is a brain region that has been implicated in a plethora of neurological and psychiatric disorders. However for a long time, its anatomical complexity has hindered a thorough investigation of the contribution of different mPFC cell-types and their afferent and efferent projections, to the development and expression of behavior associated with neural dysfunction. Through its many connections with other cortical and subcortical areas (Groenewegen et al., 1997), the mPFC may act as a control board, integrating information it receives from numerous input structures and converging updated information to output structures (Miller and Cohen, 2001). Several human psychiatric conditions, including depression, schizophrenia and substance abuse, have been linked to altered mPFC function (Tzschentke, 2001; Heidbreder and Groenewegen, 2003; Van den Oever et al., 2010). This is supported by a substantial number of experimental animal studies in which lesions, pharmacological intervention and electrophysiological techniques were employed to determine whether the mPFC is involved in cognitive processes and symptoms of psychiatric disorders (as detailed below). However, accurate dissection of the complex organization of the mPFC requires intervention with high cell-specificity and temporal resolution at a subsecond timescale. In recent years, a rapidly growing number of studies have used optogenetic approaches to address this issue, which substantially enhanced our understanding of mPFC circuitry. We will first briefly introduce the technological background and possibilities of optogenetic tools and will then review currently available literature that used optogenetics to dissect the contribution of different mPFC cell-types, and their connections within the mPFC and with other brain regions, to cognition and psychiatric disorders.

## Optogenetics technology

Optogenetics technology takes advantage of genetically encoded light-sensitive proteins, such as microbial opsins, that are introduced in intact living mammalian neurons, allowing manipulation of neuronal activity *in vitro* and *in vivo* (Boyden et al., 2005; Deisseroth, 2010). The technique is characterized by the ability to modulate neuronal firing on a millisecond timescale with great cell-type specificity in awake, freely moving animals (Gradinaru et al., 2007). A widely used depolarizing opsin is Channelrhodopsin-2 (ChR2; and genetically modified variants), a cation channel that induces action potential firing upon illumination with pulses of blue light (Mattis et al., 2012). In contrast, the chloride pump Halorhodopsin (NpHR) or the proton pump Archaerhodopsin (Arch or ArchT) are often used to hyperpolarize neuronal membranes (Mattis et al., 2012). An elaborate discussion on the use and relevance of different opsin variants and optogenetic tools would be beyond the scope of this review, but has been excellently reviewed by others (Zhang et al., 2010; Yizhar et al., 2011a). In brief, cell-type specific expression of opsins can be achieved using gene-based targeting strategies (Zhang et al., 2010). Transgenic animals and viral constructs carrying opsin genes under direct control of tissue specific promoter sequences enable the expression of opsins in genetically defined cell-types (see supplementary Table S1 for an overview of optogenetic manipulations discussed in this review). Alternatively, cell selective expression can be achieved using mouse or rat Cre-recombinase (Cre) driver lines combined with Cre-dependent viral opsin vectors. With respect to excitatory pyramidal neurons that are present in the mPFC, the CaMKIIα or Thy1 promoter can be used to express opsins in these cells (Gradinaru et al., 2007; Van den Oever et al., 2013). As these are relatively strong promoters, they are suitable to drive the expression of an opsin gene placed downstream of the promoter. Promoter regions that are used to target GABAergic interneurons are generally relatively weak promoters, and therefore modulation of mPFC interneuron activity is typically achieved using transgenic mice in which a GABAergic cell-specific promoter drives the expression of Cre (Zhang et al., 2010). For example, to manipulate fast-spiking GABAergic interneurons, parvalbumin (PV)::Cre mice are widely used (Sohal et al., 2009; Sparta et al., 2014). When these transgenic animals receive a viral vector in which the opsin gene is inserted in a double floxed inversed open reading frame, Cre expressing cells will irreversibly invert the open reading frame to enable opsin expression driven by a strong ubiquitously active promoter (e.g., elongation factor 1α; EF1α promoter) (Zhang et al., 2010).

For *in vivo* experiments, light can be delivered in the brain by a laser or LED device coupled to a thin optical fiber (~100–300 μm) that is implanted in the brain and aimed at opsin expressing cells (Sparta et al., 2012). The type of opsin used and the depth of the tissue illuminated determine the wavelength and appropriate light source required. In addition to optic modulation of opsin expressing somata, projection-specific manipulation is feasible by illuminating opsin expressing efferent projections in an innervated target region (Zhang et al., 2010). Other advantages include rapid reversibility and repeatability of photostimulation, integration with electrophysiological recordings and anatomical tracing using fluorescent reporter proteins fused to opsins (Gradinaru et al., 2007). Important limitations to consider are the toxicity of viral vectors and the potentially harmful heating of neurons during photostimulation. Albeit with few limitations, optogenetic approaches have an unprecedented capacity to selectively and robustly modulate mPFC neuronal activity in behavioral paradigms and acute slice preparations (Yizhar et al., 2011a). As the vast majority of currently published optogenetic experiments were performed in mice and rats, we will primarily focus on the anatomy and functionality of the rodent mPFC circuitry.

## Anatomy

Within the mPFC, four distinct areas have been defined along a dorsal to ventral axis, i.e., the medial precentral area (PrCm; also known as the second frontal area (Fr2)), the anterior cingulate cortex (ACC), the prelimbic cortex (PLC) and the infralimbic cortex (ILC; Heidbreder and Groenewegen, 2003). In addition to this division, which is mainly based on cytoarchitectural differences, the mPFC is often divided into a dorsal component (dmPFC), encompassing the ACC and dorsal region of the PLC, and a ventral component (vmPFC), encompassing the ventral PLC, ILC and dorsal peduncular cortex (DPC), according to functional criteria and connectivity with other brain areas (Heidbreder and Groenewegen, 2003). For the purpose of this review, in the following sections we will focus mainly on anatomical evidence derived with optogenetic tools, and mention the precise subregion of the mPFC when this information is available, or otherwise refer to dmPFC and vmPFC.

### Cytoarchitecture of the mPFC

The local mPFC network consists mainly of excitatory pyramidal cells (80–90% of the total population) and inhibitory GABAergic interneurons (10–20% of the total population), both of which can be further subdivided into different cell types based on morphological, physiological and molecular properties (Ascoli et al., 2008; DeFelipe et al., 2013). Well-studied GABAergic interneuron subtypes include the perisomatic targeting fast spiking parvalbumin (PV) interneurons, and the dendritic targeting somatostatin (SOM) interneurons. PV interneurons are of particular clinical interest, as their numbers are known to be decreased in schizophrenia patients (discussed below) (Beasley and Reynolds, 1997; Lewis et al., 2005). Both interneuron types exert strong control over local circuitry, as they are able to synchronize the spiking activity of pyramidal cells generating neuronal oscillations (Kvitsiani et al., 2013). Selective photostimulation of ChR2-expressing PV and SOM interneurons in the mPFC of mice has been shown to generate distinct circuit responses (Kvitsiani et al., 2013). Parvalbumin neurons were found to control the outputs of principal pyramidal neurons, as they exerted fast, powerful and uniform inhibition on principal cell firing (Kvitsiani et al., 2013; Sparta et al., 2014). Somatostatin neurons on the other hand modulated the input that principal pyramidal neurons received and the inhibitory effect of synchronous photostimulation of these neurons was weak, more variable and stretched over a longer time (Kvitsiani et al., 2013). Optogenetic approaches validated the critical contribution of GABAergic interneuron firing to gamma oscillations and emotional behavior (Vertes, 2006; Cruikshank et al., 2012; Yizhar, 2012; Little and Carter, 2013). Pyramidal neurons in layer V (see below) of the mPFC can be characterized as thick tufted, subcortically projecting cells and as thin-tufted, colossally projecting cells (Dembrow and Johnston, 2014). Optogenetic modulation revealed that colossally projecting cells differentially innervate both subtypes and showed that PV interneurons preferentially inhibit subcortically projecting pyramidal neurons (Lee et al., 2014a). Pyramidal cell subtypes can also be distinguished based on expression of the dopamine D1 or dopamine D2 receptor (D1-R and D2-R), of which D1-R neurons have been implicated in control over food intake by selective optogenetic activation of this population (Land et al., 2014).

### Layers and connectivity of the mPFC

The laminar organization of the rodent mPFC is slightly different from that of other cortical regions, which have a distinct input layer IV (Uylings et al., 2003). The efferent projections of granular cortices to subcortical areas arise from the deep layers V and VI, and granular cortico-cortico connections are mainly made by neurons in the superficial layers II and III (Douglas and Martin, 2004). The rodent mPFC however lacks the classical input layer IV (Uylings et al., 2003). Furthermore, both deep and superficial mPFC layers receive long-range inputs from cortical and subcortical regions and project to other (limbic) structures (Sesack et al., 1989; Gabbott et al., 2005; Hoover and Vertes, 2007).

The laminar pattern has important implications for signal processing in the mPFC. Afferent projections originating from limbic and cortical regions mainly target the superficial layers I and II/III (Romanski et al., 1999). For long, technical constraints have hampered the mapping of functional connections, as mere overlap of a spine and axonal varicosity does not necessarily indicate a functional connection and paired recordings are unsuitable for exploring long-range connections (Petreanu et al., 2007). Furthermore, most long-range excitatory inputs are severed in acute slices, hindering measurements with electrical stimulation. Optogenetic activation of ChR2-expressing presynaptic terminals showed that layer II PLC pyramidal neurons received functional inputs from the contralateral mPFC, midline thalamic nucleus (MTN), basolateral amygdala (BLA), and ventral hippocampus (HPC; Little and Carter, 2012). These input fibers synapsed at different dendritic locations, which were often poorly predicted by anatomy alone, and the connections showed bias for populations of spines of distinct volume (Little and Carter, 2012). As spine volume has been suggested to correlate with the strength of excitatory postsynaptic current (EPSC; Humeau et al., 2005), this finely tuned anatomical and functional connectivity ideally positions the mPFC to integrate and relay information from preferential afferent sources. Both dmPFC and vmPFC are heavily interconnected with the thalamus (Gabbott et al., 2005; Vertes, 2006). Thalamocortical connections are vital for mediating processes of sensation, perception, and consciousness (John, 2002; Alitto and Usrey, 2003). In addition to the thalamic input received by layer II neurons (Little and Carter, 2012), thalamic neurons that synapse onto mPFC layer I neurons have also been identified with optogenetics (Cruikshank et al., 2012). Photostimulation of thalamocortical projections originating from midline and paralaminar thalamic nuclei drove fast and robust synaptic responses in layer I late-spiking interneurons, which were more heavily excited than pyramidal cells (Cruikshank et al., 2012). These interneurons were able to drive feed forward inhibition of layer II/III pyramidal cells (Cruikshank et al., 2012). In contrast, pharmacological activation of layer I neocortical interneurons using cholinergic agonists did not induce feed-forward inhibition (Christophe et al., 2002). Furthermore, synaptic responses of mPFC interneurons were sustained upon repetitive photostimulation of thalamocortical projections (Cruikshank et al., 2012). These optogenetic findings suggest that thalamocortical projection neurons are able to drive transmission over relatively long periods of time (minutes), required for working memory function (discussed below).

The mPFC subregions are also reciprocally interconnected (Heidbreder and Groenewegen, 2003). Connectivity between ILC and PLC has been assessed by tracing methods and recently also by optogenetic tools (Vertes, 2004; Ji and Neugebauer, 2012). Ji and Neugebauer demonstrated that photostimulation of ILC pyramidal cells reduced spontaneous and evoked activity in PLC pyramidal cells, probably mediated by feed forward inhibition (Ji and Neugebauer, 2012). In contrast, both spontaneous and evoked activity in ChR2 expressing deep-layer ILC pyramidal neurons was increased upon optical activation of this neuronal population, without affecting ILC inhibitory neuron spiking behavior (Ji and Neugebauer, 2012). As the ILC and PLC project differently over the brain and have differential roles in several processes, including habitual behavior, expression of conditioned-fear and addictive behavior (Killcross and Coutureau, 2003; Vertes, 2004; Van den Oever et al., 2010; Sierra-Mercado et al., 2011), this mechanism may allow the ILC to inhibit PLC output, while simultaneously activating its subcortical target regions.

The mPFC heavily projects to other cortical and subcortical brain regions, which enables it to exert control over visceral, automatic, limbic and cognitive functions (Miller and Cohen, 2001; Hoover and Vertes, 2007). Tracing studies have shown a dorsoventral shift along the mPFC from predominantly sensorimotor target regions of the dmPFC to limbic target regions of the vmPFC (Sesack et al., 1989; Hoover and Vertes, 2007). Glutamatergic projections of the mPFC to the nucleus accumbens (NAc) core and shell have been well described and validated by optogenetic approaches (Britt et al., 2012; Suska et al., 2013). Interestingly, by microinjection of a Cre-dependent ChR2 AAV vector in Dlxi12b::Cre mice, Lee et al. (2014c) provided evidence for the existence of mPFC GABAergic neurons that have long-range projections to the NAc. This indicates that not all GABAergic neurons residing in the mPFC are local interneurons. In addition, glutamatergic PLC projections to the BLA have been studied using optogenetics technology. This pathway is thought to be important for integrating higher cognitive processing with innate emotional responses (Yizhar, 2012), a process dysregulated in mood disorders (covered in greater detail below). Little and Carter (2013) optogenetically targeted PLC layer II and identified two distinct pyramidal cell populations within this layer that either project to the contralateral mPFC or to the BLA. These PLC projection neurons were similar in anatomy and physiological properties, complicating examination of their circuit function. Photostimulation of contralateral mPFC or BLA ChR2-expressing presynaptic terminals paired with whole-cell recordings of mPFC or BLA projecting pyramidal neurons demonstrated that BLA to BLA-projecting PLC neurons exhibited the strongest synaptic connection. Enhanced synaptic transmission in this pathway was associated with increased spine density, larger spine volume and synaptic targeting. Moreover, BLA inputs targeted spines near the soma of PLC-BLA neurons, which were able to elicit stronger EPSCs than projections targeting the dendrite (Little and Carter, 2013). PLC-BLA projections also target a fraction of GABAergic interneurons in the BLA, which in some cases evoked feed-forward inhibition of GABAergic transmission (Hübner et al., 2014). This unique interconnectivity between the PLC and BLA may enable highly efficient bi-directional communication, which could be important for top-down control over responding to emotional stimuli.

These initial investigations demonstrate the unique possibilities of optogenetics to probe the mPFC circuitry at the level of individual cells, intra-mPFC connectivity and long-range afferent and efferent projections. Photostimulation in acute slice preparations is a highly relevant method to anatomically dissect functional connections and to measure synaptic properties between different neuronal populations. However, to determine whether a specific connection is causally involved in a defined cognitive process, *in vivo* modulation of neural activity is required. In the following sections, we will discuss findings derived from optogenetic interventions in freely moving animals.

## Cognition

Traditional manipulation techniques have implicated the mPFC in a diverse range of cognitive functions, of which working and long-term memory performance, alertness and habitual behavior thus far have been addressed by optogenetics technology.

### Working memory performance, alertness and temporal control

Working memory is a complex brain process that refers to temporary storage of information (time scale of seconds to minutes) necessary for cognitive performance (Baddeley, 1992). The mPFC has been implicated in this process as it was found that reversible pharmacological inactivation of the PLC impaired working memory performance (Gilmartin and Helmstetter, 2010). Working memory function can be assessed using the trace fear-conditioning task, in which a conditioned stimulus is followed by an aversive unconditioned stimulus after a delay of several seconds. Prefrontal neurons are known to exhibit persistent firing during the delay (Gilmartin and McEchron, 2005), suggesting a role for the mPFC in maintaining a representation of the conditioned stimulus during the delay. However, causal evidence for necessity of mPFC neuronal activity bridging the delay has only recently been provided using optogenetic intervention. Gilmartin et al. (2013) expressed ArchT in PLC neurons (using a non-selective CAG promoter) to allow inhibition specifically during the delay phase of the trace fear-conditioning task. Indeed, photoinhibition during the delay impaired learning of an association between the conditioned and unconditioned stimulus, confirming that spiking of PLC neurons is required for working memory performance during trace fear-conditioning. A different task to measure working memory performance is the operant delayed alternation task, in which animals alternate lever presses with a predetermined delay to obtain a reward (Dunnett et al., 1999). Excitotoxic lesions and pharmacological inactivation of the mPFC specifically impaired acquisition and expression of the delayed alternation task with long delays, indicating that mPFC activity is crucial when working memory demands are high (Rossi et al., 2012). Lesions of the ventral striatum or dorsal hippocampus, areas that are heavily connected with mPFC, did not lead to reduced delayed alternation performance. Importantly, ChR2-mediated activation of PV interneurons in the PLC selectively during the delay also significantly impaired performance in this task (Rossi et al., 2012). Together, these studies show that PLC activity is necessary for working memory performance and demonstrate that photoactivation of PV interneurons can mimic the effects of chronic lesion and pharmacological inactivation in a spatially and temporally precise manner.

Working memory function of the mPFC is modulated by several monoamine systems, including the noradrenaline and dopamine (DA) system (Rossetti and Carboni, 2005; Robbins and Roberts, 2007). During spatial working memory, extracellular noradrenaline levels increase in the mPFC and pharmacological stimulation of alpha-2A adrenoreceptors in the PLC enhanced working memory performance (Rossetti and Carboni, 2005; Ramos et al., 2006). Using optogenetics, it was found that photoactivation of ChR2-expressing noradrenergic projections from the locus coeruleus evoked persistent firing, a cellular correlate of working memory, in PLC and ACC pyramidal neurons, which was mediated through activation of presynaptic alpha1 and postsynaptic alpha2 adrenoreceptors (Zhang et al., 2013). Cortical noradrenaline has not only been implicated in working memory function, but is believed to correlate more generally with states of attention, wakefulness and arousal (Berridge, 2008). Carter et al. (2010) used optogenetic intervention to precisely evoke noradrenaline transmission and to study its influence on alertness in mice. Illumination of NpHR-expressing locus coeruleus noradrenergic neurons reduced wakefulness during the animal’s active period and caused a decrease of extracellular noradrenaline levels in the mPFC. In line with this, tonic and phasic photostimulation of ChR2-expressing locus coeruleus neurons produced immediate sleep-to-wake transitions. Interestingly, tonic activation increased general locomotor activity, whereas phasic activation had the opposite effect. Moreover, sustained high frequency (>5 Hz) photoactivation of locus coeruleus neurons evoked a state of behavioral arrest. Carter et al. (2010) show that this latter effect may be induced by a depletion of mPFC noradrenaline stores, as prolonged photostimulation reduced extracellular noradrenaline levels in the mPFC, and behavioral arrests were attenuated by noradrenaline reuptake inhibitors. This elegant study shows that prefrontal noradrenaline release is finely tuned to influence wakefulness, with even subtle differences having significant effects on sleep-to-wake transitions and arousal.

Working memory is generally considered to represent memory of two sensory stimuli that are separated by a delay. Time-tracking or memory of a defined time-interval at a timescale of seconds is thought to involve an internal clock system, in which the mPFC circuitry has also been implicated (Kim et al., 2013). In particular, DA transmission in the mPFC has been implicated in the timing of a defined interval using the fixed interval-timing task (Drew et al., 2003). In a recent study, D1-R transmission in the mPFC was shown to have a critical role in the temporal control of movement towards a goal (reward) during a defined time-interval (Narayanan et al., 2012). Pharmacological blockade of D1-R, but not the D2-R in the ILC and PLC impaired temporal control over responding in the fixed interval-timing task. In support of the specific role of D1-Rs, NpHR-mediated optical inhibition of mPFC D1-R expressing neurons impaired fixed interval timing performance (Narayanan et al., 2012). Strikingly, ChR2-mediated stimulation of D1-R neurons during the last 10 s of a 20-s interval enhanced responding only at 20 s. Based on this evidence, the authors argue that the mPFC D1 system regulates temporal control of goal-directed behavior, rather than the encoding of passage of time.

Despite considerable advances in recent years, much remains to be learned about the neurobiological substrate of working memory and related functions by comparing mPFC optogenetic interventions in different tasks within the same animal. This is of relevance to, for example, assess the commonalities and differences in mPFC circuitry mechanisms that regulate interval timing and working memory performance. Finely tuned firing of mPFC D1 neurons mediates precise temporal control over goal directed responding, but whether (sustained) activity of this neuronal population is also required for optimal working memory performance remains to be studied (Narayanan et al., 2012; Gilmartin et al., 2013). Furthermore, although traditional manipulation approaches indicate that the mPFC cholinergic system has a pivotal role in working memory (Chudasama et al., 2004), within the mPFC, this neurotransmitter system has not been directly targeted yet by optogenetics technology.

### Learning, memory and extinction

The mPFC is thought to exert cognitive control over conditioned responding to aversive and rewarding stimuli by integrating information about experienced contexts and events (Euston et al., 2012). The fear-conditioning paradigm is a widely used animal model to study learning and memory function, as well as extinction of acquired fear memories (LeDoux, 2000; Milad and Quirk, 2012; Maren et al., 2013). Specific roles for mPFC subareas have been established in the expression of conditioned fear memory, with dorsal regions mediating the encoding and expression of fear memory and ventral regions contributing to consolidation and expression of extinction memory (Peters et al., 2009; Courtin et al., 2013). These findings are supported by lesions, pharmacological inactivations and *in vivo* spike recordings (Morgan and LeDoux, 1995; Milad and Quirk, 2002; Courtin et al., 2013). However, research into the temporal contribution of specific mPFC circuitry elements has only been initiated recently. Using optogenetics, Courtin et al. (2014) established that phasic inhibition of dmPFC PV interneurons underlies the expression of fear, as assessed by freezing behavior in the fear-conditioning paradigm. They first showed that activity of a specific subpopulation of GABAergic interneurons is inhibited during the presentation of a conditioned stimulus associated with a foot-shock. Next, this subpopulation was identified as PV interneurons, since ChR2- and ArchT-mediated optical modulation of PV neurons, respectively, attenuated or evoked expression of conditioned-fear. Remarkably, optical inhibition of these neurons also evoked freezing behavior before fear-conditioning and reinstated expression of fear after extinction training (Courtin et al., 2014). They found that the PV neuron controlled fear response was mediated by resetting of theta phase oscillations in the mPFC and disinhibition of pyramidal cells projecting to the BLA, further supporting the role of the mPFC-BLA projection in emotional control. This study also identified a second population of inhibitory interneurons that showed increased activity during fear states. The authors speculate that this subpopulation may inhibit PV interneurons and receives input from brain regions (e.g., hippocampus, BLA) that drive the expression of fear (Courtin et al., 2014), an interesting hypothesis that remains to be addressed by future research. Extinction of conditioned-fear is associated with decreased excitatory synaptic efficacy transmission of mPFC to BLA pyramidal cells, but did not affect output to GABAergic BLA interneurons and intercalated cells, as demonstrated using optogenetics (Cho et al., 2013). As a result, the excitation/inhibition (E/I) balance in this pathway is likely changed, favoring inhibition and resulting in suppression of the conditioned-fear response (Cho et al., 2013). These optogenetic studies confirm the role of the dmPFC in driving of fear responses and refine the temporal contribution of subpopulations of GABAergic interneurons in this behavior. An interesting study by Lee et al. (2014c) showed that photoactivation of long-range GABAergic mPFC projections to the NAc evoked real-time place avoidance, suggesting this novel pathway may also regulate responding to aversive stimuli.

### Habitual behavior

Habits are defined as behavioral patterns that are insensitive to changes in outcome value. Habitual behavior is differentially regulated by mPFC subareas; whereas the PLC promotes flexibility, ILC activation inhibits flexibility and promotes behavioral rigidity (Killcross and Coutureau, 2003). Previous studies demonstrated that lesion and pharmacological inactivation of the ILC induce a switch from fixed to flexible responding (Coutureau and Killcross, 2003). The temporal control of ILC neurons to habitual behavior has been confirmed and refined by repetitive optogenetic modulation. Brief photoinhibition of ILC pyramidal cells blocked the formation and expression of habitual behavior, but the subsequent behavioral response depended on the timing of inhibition (Smith et al., 2012; Smith and Graybiel, 2013). In these studies, habitual behavior was assessed by training rats to obtain a reward in a cued T-maze task. Following overtraining, rats became insensitive to devaluation of the reward. Animals continued goal-directed behavior when ILC pyramidal cells were optogenetically silenced during habit formation, but once the habit was fully expressed, photoinhibition evoked a new habitual pattern. Moreover, when photoinhibition was repeated during execution of the new habit, animals re-expressed the original habit (Smith et al., 2012). This immediate switching between habitual behaviors demonstrates that even semiautomatic behaviors are under cortical control while they are being performed. The ILC target region that mediates switching between habits has not been identified yet, but projections to the dorsolateral striatum are of particular interest, as a similar spike activity pattern was observed in both regions after a habit was established (Smith and Graybiel, 2013). Based on this evidence, the authors suggested that the development of habitual performance is determined by the balance of sensorimotor striatal activity and value-sensitive ILC activity. Interestingly, only the superficial ILC layers mimicked spiking activity in the dorsolateral striatum (Smith and Graybiel, 2013), stressing the need to apply layer- and pathway-specific optogenetic manipulations to study the habit circuitry in more detail.

## Psychiatric disorders

Optogenetics provided important new insights in mPFC function in the healthy brain, but has also been used to elucidate neural circuitry elements involved in disease-related phenotypes (Steinberg et al., 2014). In the following sections, we will discuss how optogenetic manipulations have validated, and in some cases updated current theories that aim to explain the contribution of the mPFC circuitry to various psychiatric disorders, including depression, schizophrenia and drug addiction.

### Depression

Major Depressive Disorder (MDD) is one of the most prevalent psychiatric disorders, estimated to affect about 5% of the global population and therefore considered as a leading cause of disability worldwide (World Health Organization, 2012). Major Depressive Disorder diagnosis criteria include depressed mood and anhedonia (reduced ability to experience pleasure) that persist over time and affect every day-life experience (American Psychiatric Association, 2013). In addition, MDD diagnosis includes somatic effects, such as disturbances in food intake (weight loss or gain), in sleep (insomnia or hypersomnia), as well as in levels of psychomotor activity (agitation or retardation). Cognitive decline characterized by impairments in working memory and decision making, loss of concentration and attentional biases is also considered a key factor in perpetuation of the depressive state (Murrough et al., 2011). The multifaceted phenotypic expressions that accompany depression are attributed to dysfunctional processes in multiple brain areas and circuitries, including the brain’s reward, affective and executive control centers.

As the mPFC is considered a circuit hub that promotes higher-order cognitive functions and provides top-down control over automatic limbic system-associated processes (Clark et al., 2009; Murrough et al., 2011; Treadway and Zald, 2011), it is suggested to have critical role in affective and cognitive deficits associated with depression. In humans, depressive states are linked to disrupted frontal activity (hyper- or hypo-activation) and morphology, which are thought to contribute to working memory deficits, maladaptive regulation of emotions (anhedonia, negative affect), attentional biases and impaired decision making (Southwick et al., 2005; Fales et al., 2008; Beevers et al., 2010; Disner et al., 2011). Stress exposure, tightly associated with the onset and development of the depressive state, is considered detrimental for mPFC functioning. Proper mPFC performance is necessary for modulating stress-induced behavioral adaptations and for exerting control over stress-activated subcortical regions (Amat et al., 2005; Czéh et al., 2007; Arnsten, 2009; Dias-Ferreira et al., 2009; Treadway et al., 2013). In recent years, the clinical toolbox for treating depression has been expanded with deep brain stimulation (DBS) of the PFC. These recent studies showed that chronic stimulation of the subgenual cingulate cortex (Cg25), the human equivalent of the rodent vmPFC (Hamani et al., 2010b; Chang et al., 2014), reverses depression-induced cortical functional deficits and alleviates symptoms in treatment-resistant depressed patients (Mayberg et al., 2005). Subsequent reverse translational studies confirmed the involvement of the mPFC in antidepressant-like responses, as high-frequency electrical stimulation of the rat PLC alleviated behavioral despair modeled in the forced swim test (FST; Hamani et al., 2010a), which correlates with motivational, active adaptation to challenging environments. Similarly, following chronic unpredictable mild stress, chronic vmPFC DBS reduced depression-associated anhedonia, as assessed by a sucrose preference test in rats and relieved from social avoidance in mice susceptible to chronic social defeat stress (Hamani et al., 2012; Veerakumar et al., 2014). Taken together, over the years both clinical and preclinical research implicated the mPFC as a crucial mediator of depressive symptomatology (Koenigs and Grafman, 2009), which triggered a quest for causality and a clarification of the exact contributions of mPFC subregions and their distinct afferent and efferent projections in the development of the disorder and antidepressant response.

**Figure 1:**
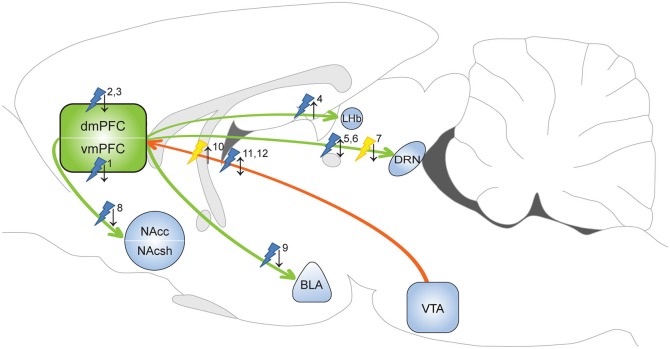
**Optogenetic evidence for the involvement of the mPFC in depressive-like behavior and anxiety**. Yellow flash: photoinhibition; blue flash: photoactivation; ↑ = pro-depressive/anxiogenic effects; ↓ = antidepressant/anxiolytic effects. ^1^Covington et al. (2010): photoactivation increased sucrose preference and restored social interaction in defeat-susceptible mice. ^2^Kumar et al. (2013): photoactivation layer V pyramidal cells decreased immobility FST in naïve animals. ^3^Kumar et al. (2013): photoactivation layer V pyramidal cells increased time in open arms EPM test in defeated animals. ^4^Warden et al. (2012): photoactivation of mPFC-LHb projection promoted immobility FST in naïve animals. ^5^Warden et al. (2012): photoactivation of mPFC-DRN projection decreased immobility FST in naïve animals. ^6^Challis et al. (2014): photoactivation of vmPFC-DRN projection reduced social interaction in naïve animals. ^7^Challis et al. (2014): photoinhibition of vmPFC-DRN projection prevented social withdrawal in defeated animals. ^8^Vialou et al. (2014): photoactivation of dmPFC-Nac projection prevented social withdrawal. ^9^Vialou et al. (2014): photoactivation of dmPFC-BLA projection increased time in open arms EPM test. ^10^Chaudhury et al. (2013): photoinhibition of VTA-mPFC DA projection reduced social interaction in sub-threshold defeat animals. ^11^Friedman et al. (2014): photoactivation of VTA-mPFC DA projection restored social interaction in defeat-susceptible mice. ^12^Gunaydin et al. (2014): photoactivation of VTA-mPFC DA projection evoked anxiety-like behavior and place avoidance in naïve mice. dmPFC: dorsal medial prefrontal cortex; vmPFC: ventral medial prefrontal cortex; NAcc: nucleus accumbens core; NAcsh: nucleus accumbens shell; LHb: lateral habenula; DRN: dorsal raphe nucleus; BLA: basolateral amygdala; VTA: ventral tegmental area.

The first optogenetic experiments that directly assessed the role of mPFC activity in depression-like behavior confirmed that activation of vmPFC neurons reverses depressive-like symptomatology in a depression-vulnerable population of mice (Covington et al., 2010; Figure 1). In this study, the authors used the chronic social defeat paradigm, a depression model with high face, predictive and construct validity (Nestler and Hyman, 2010) to distinguish mice on their resilience/vulnerability to social stress. Photostimulation of the vmPFC was achieved using a herpes simplex virus (HSV) viral vector coding for ChR2 driven by the IE4/5 promoter, which targeted ChR2 to mPFC neurons in a non-selective manner (Covington et al., 2010). Specifically, the ILC and PLC of stress-susceptible mice were stimulated in a pattern similar to DBS parameters that previously alleviated depressive symptoms, mimicking cortical burst firing (Hamani et al., 2010a). Photostimulation fully restored social interaction scores and diminished anhedonia, as expressed in preference for drinking a sucrose solution over water, without altering anxiety levels or social memory performance (Covington et al., 2010). Notably, traditional mPFC manipulations have led to contradictory observations. For example, generic mPFC lesions led to the expression of depressive-like behavior, including learned helplessness (Klein et al., 2010), whereas transient pharmacological inactivation of the ILC resulted in an antidepressant response, as assessed by the FST (Slattery et al., 2011). These opposing findings might originate from the different temporal resolution of the methodologies and/or the different (sub) regions examined, e.g., whole mPFC (Klein et al., 2010) vs. vmPFC (Covington et al., 2010) or ILC (Slattery et al., 2011). As optogenetic activation of the vmPFC by Covington et al. (2010) was not specific for a particular neuronal subtype, the direction of the net effect of stimulation at the circuit level remains unresolved. These data may reflect the variability of mPFC involvement seen in human studies, which support either reduced or increased activity of distinct frontal areas in the expression of the depressive state.

In a subsequent study, Kumar et al. (2013) employed layer V pyramidal cell-specific photostimulation of the PLC to examine the contribution of this mPFC sub-region in depressive-like symptomatology. To this end, Thy1::Chr2 mice expressing ChR2 in pyramidal cells projecting to limbic structures, including the ventral tegmental area (VTA), BLA and NAc were used. Acute PLC stimulation in naïve animals induced a robust antidepressant-like response, as expressed in reduced immobility in the FST. Accordingly, in animals subjected to the chronic social defeat model, chronic optical stimulation of PLC pyramidal cells induced a long-lasting anxiolytic effect in the elevated plus maze (EPM) test, a classical test to assess anxiety. In addition to the behavioral effects of PLC stimulation, the authors reported synchronized oscillatory activity across PLC target limbic structures (VTA, BLA and NAc), providing evidence for downstream effects of PLC pyramidal cell modulation on subcortical regions responsible for affective and reward-related processing. Importantly, similar alterations in neuronal activity in this circuit has been observed in depressed patients (Sheline et al., 2010) and might underlie the antidepressant-like effects of mPFC DBS in humans (Mayberg et al., 2005). Interestingly, in contrast to vmPFC activation, PLC pyramidal cell stimulation did not reverse the well characterized defeat-induced social avoidance phenotype (Kumar et al., 2013). These discrepancies may be attributed to the different frequency stimulation parameters used or the different cell-types and mPFC layers targeted. Importantly, as the optic fiber in these experiments was targeted to ChR2+ somata in the mPFC, the exact projections that exerted the antidepressant-like effects remain to be determined by projection-specific targeting.

Warden et al. examined the role of mPFC efferents in depressive behavior, with a focus on projections to the dorsal raphe nucleus (DRN) and the lateral habenula (LHb; Warden et al., 2012), regions that are heavily implicated in MDD (Sartorius et al., 2010; Willner et al., 2013; Albert et al., 2014; Mahar et al., 2014). The mPFC-DRN projection is of particular interest, as the antidepressant effect of vmPFC DBS in rats is accompanied by structural and functional alterations in serotoninergic DRN neurons (Veerakumar et al., 2014) and it is completely abolished following serotoninergic depletion in the DRN (Hamani et al., 2012). In naïve animals, optogenetic activation of the mPFC-DRN excitatory projection through illumination of mPFC terminals in the DRN promoted behavioral activation in the FST (Warden et al., 2012). In contrast, photoactivation of mPFC terminals in the LHb induced immobility in the FST, whereas illumination of vmPFC pyramidal cell bodies was without effect. More recently, the vmPFC-DRN pathway contribution to a depressive-like state was examined using the chronic social defeat paradigm (Challis et al., 2014). In naïve animals, repeated ChR2-mediated activation of vmPFC-DRN projections increased avoidance of a social target, pointing to a depressive-like phenotype. In line with this, Arch-mediated photoinhibition of the same pathway prevented the development of social withdrawal in animals subjected to social defeat (Challis et al., 2014). The authors provide evidence that vmPFC neurons mainly target GABAergic neurons in the DRN, which likely inhibit serotonergic neurons, explaining the pro-depressant effects they observed. However, their data is inconsistent with the anti-depressive, proactive effects that were found in the FST following stimulation of the vmPFC-DRN pathway (Warden et al., 2012). This suggests that the mPFC-DRN pathway may be differentially involved in regulating social interaction and behavioral despair, the two behavioral constructs these tests assess. Alternatively, the contrasting observations may be explained by a differential effect of acute (Warden et al., 2012) vs. repeated post-defeat photoactivation of the vmPFC-DRN pathway (Challis et al., 2014) on expression of depressive-like behavior. Nonetheless, these experiments demonstrate the contributions of the mPFC to the adaptive capacity under physically (proactive vs. passive reactivity) or emotionally (affective decision-making) challenging conditions, which is severely disrupted in depression (Gotlib et al., 2008; Derntl et al., 2011; Volman et al., 2011; Cruwys et al., 2014). Vialou et al. (2014) showed that PLC-NAc and PLC-BLA projections are differentially involved in depression susceptibility and anxiety-related behavior. They found that chronic social defeat stress up-regulated ΔFosB in the PLC, which was linked to increased cholecystokinin B (CCKB) receptor expression and the induction of a depression-susceptible phenotype in animals exposed to sub-threshold defeat stress (Vialou et al., 2014). In support of this, local application of a CCK agonist (CCK-8) in the PLC promoted a susceptible phenotype and ChR2-mediated optical stimulation of PLC glutamatergic terminals in the NAc prevented CCK-8 administration-induced social deficits (Vialou et al., 2014). CCK-8 infusion in the PLC also produced an anxiogenic effect in the EPM and this effect was reversed by photostimulation of the PLC-BLA, but not PLC-NAc, pathway. Taken together, these data highlight the importance of selectively manipulating specific mPFC projections to determine their role in top-down control of subcortical structures in depressive-like behavior and (mal) adaptive responsiveness to stressors (Lobo et al., 2012; Yizhar, 2012; Shenhav and Botvinick, 2013).

In addition to the modulation of efferent projections, optogenetics has also been used to intervene with mPFC afferent DA projections (Chaudhury et al., 2013; Friedman et al., 2014; Gunaydin et al., 2014). To selectively manipulate the VTA-mPFC DA projection, Chaudhury et al. (2013) microinjected a retrograde traveling pseudorabies virus coding for Cre in the mPFC and Cre-dependent ChR2 or NpHR vectors in the VTA. Photoinhibition of the VTA-mPFC pathway reduced social interaction in mice that underwent sub-threshold social defeat (Chaudhury et al., 2013). Interestingly, they also found that the firing rate of VTA DA neurons that project to the mPFC was substantially reduced in susceptible mice that received social defeat stress. Together, this indicates that DA release in the mPFC may prevent the development of a depression susceptible phenotype. Channelrhodopsin-2-mediated activation of the VTA-mPFC pathway did not affect the development of a susceptible phenotype following sub-threshold social defeat (Chaudhury et al., 2013). However, repeated stimulation of ChR2-expressing VTA-mPFC neurons reversed social avoidance in a depression-susceptible population following chronic social defeat (Friedman et al., 2014). Opposite effects have been observed of ChR2-mediated stimulation of the VTA-mPFC DA pathway in naïve mice, which showed no change in social interaction, but instead showed an increase in anxiety-like behavior and conditioned place aversion (Gunaydin et al., 2014). Together, these studies demonstrate that the direction of behavioral effects depends on the behavioral state of an animal. In depression-prone animals, alterations in the activity of mPFC afferent DA projections are sufficient to enhance vulnerability to develop a depressed phenotype or to reverse depressive-like behavior.

Optogenetic control of the mPFC and connected brain regions has greatly advanced our understanding of the neurobiological underpinnings of depression (Lammel et al., 2014). In particular, important steps have been made in the dissection of the contribution of specific mPFC efferent projections to specific behavioral components of the depressive symptomatology, such as social, anxiety and reward-related behaviors. Interestingly, these studies have also revealed resilience mechanisms, including anatomical (VTA-mPFC DA projection) and molecular (CCK) pathways, which could prove of great use in the battle against this debilitating disorder. In the future, profiling of gene and protein expression changes in the mPFC upon optogenetic stimulation could provide insight in molecular mechanisms underlying susceptibility and resilience to depressive behavior and may open new avenues for medical intervention (Lobo et al., 2012).

Despite these advances that have been made possible by optogenetic tools, several clinically relevant issues have not been addressed yet. As depression is characterized by individual-based phenotypic expression, with versatile symptomatology, single-construct assessment of depressive-like behavior and anxiety using relatively simplistic behavioral assays (FST, EPM, sucrose preference) may restrict the translational value of these findings (Belzung et al., 2014), arguing for the development and use of models with enhanced validity to study a depressed state. Importantly, cortical manipulations that affect social interaction in animals do not necessarily reflect a depressive-like phenotype, but may be indicative of mechanisms supporting social behavior in general. As such, identified mPFC circuits might also have a role in other psychiatric conditions that are characterized by social impairments, e.g., autism-spectrum disorders, anxiety disorders and schizophrenia (see below; Yizhar, 2012; Allsop et al., 2014). In addition, depending on the behavioral read-out (e.g., sociability or anhedonia), optogenetic intervention can have a differential effect (Albert, 2014), further complicating interpretation of the role of specific circuitry elements in a complex behavioral state. Finally, perturbation of the circuitries mediating depression-induced cognitive decline, which is a critical vulnerability factor for the perseverance of the disorder, has remained an unexplored area regarding optogenetic manipulations, but holds high promise for elucidation of novel targets that can be used for treatment of this prevalent psychiatric disorder.

### Schizophrenia

Schizophrenia is characterized by highly heterogeneous cognitive (working memory, attention), positive (delusions, hallucinations) and negative (flat affect, anhedonia) symptoms, as well as disorganized speech and abnormal motor behavior (American Psychiatric Association, 2013). Current pharmacotherapy addresses only a small fraction of symptoms, with the majority of treatments being limited in controlling psychosis-related deficits and unable to attend to the primary cause of disability, i.e., cognitive decline (Ross et al., 2006; Cho and Sohal, 2014). As the pathogenesis of schizophrenia remains unclear and likely involves a complex neural circuitry, optogenetic dissection of the underlying neural substrates and neuroadaptations will be instrumental for understanding this severe and currently incurable mental disorder (Peled, 2011; Cho and Sohal, 2014).

Many of the cognitive deficits accompanying schizophrenia, such as impaired working and episodic memory and impaired affective control and reward evaluation, have been traced back to dysregulated PFC function, resulting in altered connectivity with subcortical areas, such as the amygdala, striatum and the hippocampus (Ross et al., 2006; Meyer-Lindenberg, 2010; Arnsten et al., 2012). Several theories exist concerning mPFC alterations that cause schizophrenia symptoms, including altered dopaminergic modulation, a change in E/I balance and abnormal oscillatory activity in the gamma frequency range (Meyer-Lindenberg, 2010; Lisman, 2012). Optogenetic approaches have begun to address the merits of these theories by providing causal insight in the underlying mechanisms of the heterogeneous symptoms of schizophrenia, in particular the cognitive dysfunction and aberrant information processing associated with this disorder (Wang and Carlén, 2012; Touriño et al., 2013).

A dual role of dopamine has been hypothesized to contribute to the development of schizophrenia. In particular, it is thought that increased DA transmission in the mesolimbic system and parallel DA hypoactivity in the mPFC account for the expression of schizophrenic symptoms (Brisch et al., 2014; Cho and Sohal, 2014). Additionally, imbalanced activation of cortical D1-Rs and D2-Rs, which have opposing effects on neuronal excitability (Beaulieu and Gainetdinov, 2011), is considered crucial for impaired information processing and the manifestation of both positive and negative symptoms in schizophrenia (Seamans and Yang, 2004; Durstewitz and Seamans, 2008; Brisch et al., 2014). The involvement of D2-Rs is supported by the fact that all antipsychotics that are being used to treat positive symptoms of schizophrenia, block D2-R function (Cho and Sohal, 2014). Furthermore, prefrontal D2-Rs have a critical role in cognitive processes that are disrupted in schizophrenia, including working memory and sensorimotor gating, as determined with mutant mice and pharmacological interventions (Ralph et al., 1999; Seamans and Yang, 2004; Durstewitz and Seamans, 2008). Optogenetic modulation of D2-R expressing neurons in the mPFC provided new insight in the functionality of D2-Rs and their potential contribution to schizophrenia symptoms. Intra-mPFC infusion of a Cre-dependent ChR2 vector in D2-R::Cre mice enabled robust expression of ChR2 in a subpopulation of layer V pyramidal cells projecting to the thalamus (Gee et al., 2012). Acute slice recordings demonstrated that, at baseline, the D2-R agonist quinpirole had minimal effect on current injections in D2-R neurons, however, a significant after-depolarization occurred when quinpirole application was closely preceded by optogenetic activation of contralateral D2-R-expressing mPFC projection neurons, generating voltage fluctuations and spiking for hundreds of milliseconds (Gee et al., 2012). Given the specificity of D2-R expression in cortico-thalamic projecting layer V neurons, D2-R-mediated after-depolarization might enhance outputs to subcortical structures. Under pathological conditions, such as D2-R overrepresentation seen in schizophrenia (Seeman and Kapur, 2000), this sustained signal amplification might enhance the level of noise in the mPFC, thereby distorting relay of information to subcortical areas and potentially enhancing susceptibility to psychosis. As the level of noise within the mPFC is thought to be increased in schizophrenic patients (discussed below), diminishing the D2-R-mediated after-depolarization might be a neurophysiological basis for the beneficial effect of antipsychotics on schizophrenia symptoms. Further research using *in vivo* models will have to verify whether D2-R induced after-depolarization is involved in the cognitive dysfunction observed in schizophrenia.

The E/I balance theory poses that an elevation in the ratio of cortical E/I, mediated either via hyperexcitability of pyramidal cells or hypoactivity of inhibitory interneurons, underlies the behavioral and cognitive symptoms of schizophrenia, including social dysfunction (Lisman, 2012; Wang and Carlén, 2012). Network and behavioral effects of an altered E/I balance in the mPFC has been addressed using the stable step function opsin (SSFO), a ChR2 mutant with significantly reduced deactivation time (~30 min) (Yizhar et al., 2011b; Yizhar, 2012) upon excitation with a single pulse of blue light, thereby reducing the threshold for action potential firing in SSFO-expressing neurons. Brief photoactivation of SSFO-expressing mPFC pyramidal neurons increased the E/I balance, impaired information processing at the cellular level and increased rhythmic high-frequency activity, resembling clinical indications of schizophrenia (Yizhar et al., 2011b) (see section below). At a behavioral level, these manipulations were sufficient to completely abolish social interaction and reversibly impaired acquisition of conditioned-fear memory. Enhanced E/I balance in the primary visual cortex did not alter social behavior, which alludes to specificity of the mPFC in mediating these behavioral deficits. Interestingly, depolarization of SSFO-expressing mPFC GABAergic PV neurons did not affect social interaction and conditioned-fear (Yizhar et al., 2011b), despite the fact that it robustly reduced spiking and synaptic activity. However, social deficits observed after photoactivation of SSFO-expressing pyramidal cells were partially rescued by co-activation of ChR2-expressing PV neurons (Yizhar et al., 2011b). As discussed earlier, inhibition of mPFC PV neurons can result in severe working memory deficits (Rossi et al., 2012), further stressing the importance of a properly balanced cortical excitatory tone. Notably, an elevated E/I balance within the mPFC is also thought to contribute to social dysfunction associated with autism spectrum disorders (Yizhar et al., 2011b), hence, these findings may point to a pathophysiological mechanism that mediates general impairments in social behavior. Although the use of SSFOs aids in explaining the consequence of a distorted mPFC E/I balance at a cellular level and on social interaction, altered E/I balance in schizophrenia and autism is likely the result of an aberrant neurodevelopmental mechanism. Hence, in patients, E/I balance is elevated for a time-period that is far beyond the deactivation time-scale of currently available SSFOs. The relatively “acute” effects of a change in E/I balance in developmentally normal animals should therefore be interpreted with caution. That being said, optogenetic manipulations using SSFOs have for the first time demonstrated robust differential effects of an alteration in mPFC E/I balance on network activity and behavior. Furthermore, SSFOs can be used to assess whether E/I balance is perturbed in other psychiatric diseases, including autism, depression and addiction, potentially unifying the etiology of these disorders (Tye and Deisseroth, 2012).

A third avenue that aims to explain the cognitive deficits of schizophrenia patients involves gamma rhythms, 30–80 Hz neuronal oscillations that play a pivotal function in synchronizing neuronal activity within and between areas, which is known to be required for working memory, perception and attention (Lewis et al., 2005; Wang and Carlén, 2012), and is likely important for many other brain functions. In schizophrenia patients, abnormal gamma oscillations have been consistently observed, and they correlate with changes in working memory and cognitive control (Uhlhaas et al., 2008; Uhlhaas and Singer, 2010). When PV neuron function is impaired, suboptimal inhibitory drive leads to desynchronization, contributing to altered gamma rhythm and presumably to working memory impairments associated with schizophrenia (Lewis et al., 2005). In accordance with this notion, local GABA synthesis and reuptake are consistently reduced in the PFC of schizophrenia patients and this change is specifically mediated by PV neurons, implying aberrant functionality of this particular interneuron population (Lewis et al., 2005). Similarly, reduced PV immunoreactivity in the PFC of schizophrenic patients has been reported (Beasley and Reynolds, 1997). Optogenetic studies validated the critical importance of cortical PV interneurons in driving gamma oscillations (Cardin et al., 2009; Sohal et al., 2009). Sohal et al. (2009) showed that photostimulation of ChR2-expressing PFC pyramidal cells elicited gamma oscillations *in vivo*, however, simultaneous NpHR-mediated inhibition of PV+ interneurons specifically suppressed gamma power, suggesting that pyramidal cells stimulation activated downstream PV neurons. Importantly, when subjecting pyramidal neurons to gamma-frequency input, microcircuit signal transmission was improved by reducing circuit noise and amplifying circuit signals, including signals to local interneurons (Sohal et al., 2009). Parvalbumin interneuron-driven gamma-mediated neuronal synchrony dependents on NMDA receptor activation, as targeted NMDA receptor deletion in PV neurons impaired optogenetic induction of gamma oscillations and resulted in selective cognitive decline, resembling schizophrenic deficits (Carlén et al., 2012). Together, selective optogenetic modulation of PV interneuron activity confirmed that this neuronal subtype drives gamma oscillations, which sequentially promotes fast and targeted information processing; a “sharpening” of cortical response to sensory inputs (Wang and Carlén, 2012). Changes in oscillation synchrony are also thought to underlie other psychiatric conditions, including bipolar disorder and autism, as well as epilepsy (Uhlhaas and Singer, 2006; Sheline et al., 2010). Thus, efforts aimed at further elucidation of circuit and molecular adaptations that contribute to aberrant generation of neuronal oscillations are of utmost importance.

Taken together, the first optogenetic manipulations of the mPFC circuitry have at least partially validated existing theories that aim to explain neuropathological mechanisms underlying schizophrenia. Enhanced excitatory drive, potentially as a result of D2-R overexpression, resulting in desynchronized neuronal transmission and impaired cortical information processing contributes to symptoms associated with this disorder. Given the multifaceted and complex nature of schizophrenia, it will likely be impossible to mimic the full phenotypic spectrum in an animal model. Although optogenetic manipulations in the rodent brain are invaluable for providing new directions into this field of research, the translational value of the observed mechanisms remains a challenge that needs to be addressed in the future.

### Addiction

Addicted individuals display a behavioral repertoire restricted to repeated cycles of drug seeking, consumption and recovery from drug use despite often severe negative consequences (Hyman, 2005). Drug addiction is the endpoint of a series of transitions from initial, hedonic drug use to habitual and ultimately compulsive drug use, which coincides with long-lasting adaptations in neural circuits (Robinson and Berridge, 1993; Kalivas and Volkow, 2005). High relapse rates are a major problem in treatment of addiction, as addicted individuals remain highly susceptible to relapse even after long periods (months to years) of abstinence (Kalivas and O’Brien, 2008). This persistent vulnerability is thought to be maintained by strong and persistent associative memories of drug effects and environmental cues (Hyman et al., 2006). The brain circuitry that supports addiction is complex, but ample evidence indicates that the mPFC has a significant role in the development and persistence of addictive behavior (Kalivas, 2009). More specifically, the mPFC has been implicated in the attribution of salience to rewarding stimuli, compulsive drug taking, the expression of drug-associated memories and relapse to drug seeking (Van den Oever et al., 2010; Hogarth et al., 2013; Peters et al., 2013). Optogenetic approaches confirmed the important function of the mPFC in animal models of addictive behavior and provided interesting new insights in the temporal contribution of mPFC subregions and projections to the NAc to compulsive drug taking and drug seeking behavior.

**Figure 2:**
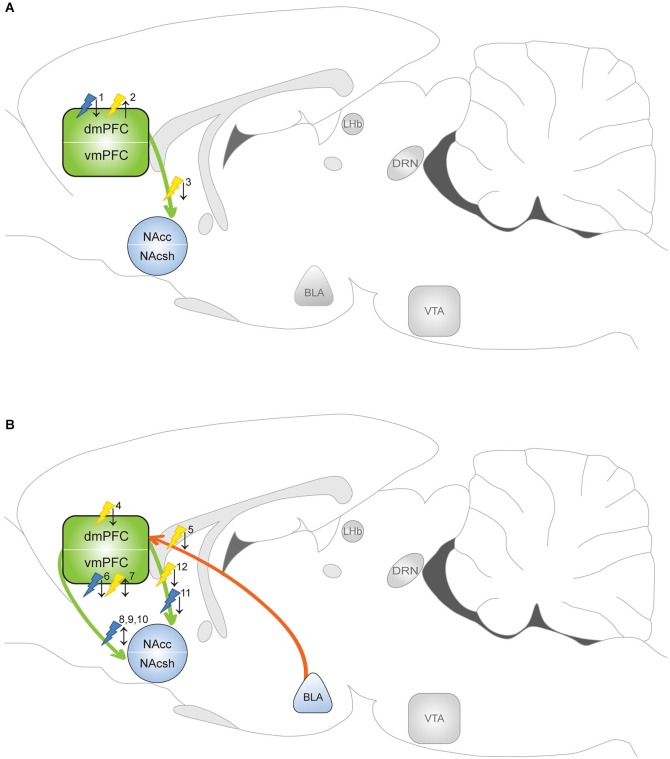
**Optogenetic evidence for the involvement of the mPFC in addictive behavior**. Yellow flash: photoinhibition; blue flash: photoactivation. ↑ = enhanced drug taking/seeking; ↓ = reduced drug taking/seeking. Optogenetic manipulations indicate that the circuitry that regulates drug taking (when the drug is available) differs from the circuitry that mediates drug seeking (in absence of the drug). **(A)** Manipulation of drug *taking*. ^1^Chen et al. (2013): photoactivation PLC diminished compulsive cocaine taking in aversion resistant rats. ^2^Chen et al. (2013) and Martín-García et al. (2014): photoinhibition PLC evoked compulsive cocaine taking in aversion sensitive rats and resumption of cocaine intake in rats with history of high-frequency self-administration. ^3^Seif et al. (2013): photoinhibition of dmPFC-NAcc projection reduced alcohol intake paired with aversive stimulus. **(B)** Manipulation of drug *seeking*. ^4^Stefanik et al. (2013) and Martín-García et al. (2014): photoinhibition dmPFC attenuated cocaine seeking. ^5^Stefanik and Kalivas (2013): photoinhibition of BLA-dmPFC projection reduced reinstatement of cocaine seeking. ^6^Van den Oever et al. (2013): photoactivation vmPFC facilitated extinction of remote, but not recent, cocaine memory. ^7^Van den Oever et al. (2013): photoinhibition vmPFC impaired recall of recent cocaine memory, but prevented extinction of remote cocaine memory. ^8^Ma et al. (2014): photoactivation (1 Hz) evoked LTD in vmPFC-NAcsh projection reversed cocaine-induced synaptic adaptation and enhanced subsequent cocaine seeking. ^9^Pascoli et al. (2012): photoactivation (1 Hz) of vmPFC-NAcsh projection reversed cocaine-induced synaptic adaptation and locomotor sensitization. ^10^Pascoli et al. (2014): photoactivation (13 Hz) of vmPFC-NAcsh projection reversed cocaine-induced synaptic adaptation and abolished cocaine seeking. ^11^Ma et al. (2014): photoactivation (1 Hz) evoked LTD in dmPFC-NAcc projection reversed cocaine-induced synaptic adaptation and decreased subsequent cocaine seeking. ^12^Stefanik et al. (2013): photoinhibition of PLC-NAc core projection attenuated cocaine-primed reinstatement of cocaine seeking.

Evidence from neuroimaging studies suggests that hypofunction of the mPFC contributes to a loss of control over limiting intake in human addicts (Goldstein and Volkow, 2011). This hypothesis was recently addressed using optogenetics in rats that continued to self-administer cocaine despite the pairing of cocaine reward with delivery of a noxious stimulus (foot-shock). Chen et al. (2013) showed that long-term cocaine self-administration reduced PLC neuron excitability, with the most robust effect in aversion-resistant rats. Restoring PLC pyramidal function by optogenetic stimulation alleviated cocaine intake in aversion-resistant rats (Figure 2A). In contrast, when PLC neurons were optogenetically silenced, non-resistant rats engaged in cocaine self-administration paired with a foot-shock. This study indicates that when cocaine use is paired with an adverse consequence, hypoactivity of PLC pyramidal cells contributes to a loss of inhibitory control over compulsive cocaine intake.

Pharmacological interventions in animal models of conditioned drug seeking indicate that the dmPFC and vmPFC differentially contribute to the expression of this specific behavior (Peters et al., 2009; Van den Oever et al., 2010). Whereas dmPFC activity is thought to drive drug seeking responses, the vmPFC either promotes or inhibits drug seeking responses depending on the type of drug that was previously administered and the implementation of extinction sessions before a drug-seeking test (McLaughlin and See, 2003; Peters et al., 2008; Rogers et al., 2008; Koya et al., 2009; Willcocks and McNally, 2013; Lubbers et al., 2014). In fact, several lines of evidence suggest that the ILC mediates the consolidation and expression of extinction memory (Peters et al., 2008; LaLumiere et al., 2010), and as such, inhibition of this region after extinction learning evokes expression of the original cocaine seeking response. Optogenetic manipulation of the vmPFC extended these findings by showing that vmPFC pyramidal cells indeed contribute to expression and extinction of conditioned cocaine seeking, but in a time-dependent manner (Van den Oever et al., 2013; Figure 2B). Channelrhodopsin-2-mediated activation of vmPFC pyramidal cells facilitated extinction of a cocaine conditioned place preference (CPP) memory only when photostimulation was applied 3 weeks after, but not 1 day after conditioning. In line with this observation, NpHR-mediated inhibition of these neurons blocked extinction of CPP memory 3 weeks after conditioning. Surprisingly, photoinhibition selectively attenuated expression of a 1 day-old cocaine memory. Together, optogenetic manipulation of pyramidal cells pointed to a temporal reorganization of the circuitry that controls expression of cocaine-associated memories and a differential role of the vmPFC in regulation of conditioned cocaine seeking over time.

Optogenetic studies confirmed that PLC activity is required for reinstatement of cocaine seeking in extinguished animals. Similar to pharmacological inactivation, photoinhibition of PLC neurons (using a non-selective promoter) reduced cocaine-primed reinstatement of cocaine seeking (Stefanik et al., 2013). In addition, the same group demonstrated that the BLA-PLC pathway is critically involved in reinstatement of cocaine seeking by optical inhibition of BLA presynaptic terminals in the PLC (Stefanik and Kalivas, 2013). Optogenetic inhibition of dmPFC pyramidal neurons also attenuated stress-induced reinstatement of palatable food seeking in rats (Calu et al., 2013), suggesting that different modalities activate dmPFC circuitry to evoke reinstatement of reward seeking. In addition, this shows that PLC activity drives reinstatement of cocaine and natural reward seeking, whereas increased activity of the same neurons suppresses compulsive cocaine taking (Chen et al., 2013). The opposing function the PLC may depend on the presence or absence of cocaine in operant tests. This is supported by the observation that photoinhibition of PLC pyramidal cells enhanced cocaine self-administration and attenuated reinstatement of cocaine seeking in rats that were subjected to a high-frequency cocaine intake schedule (Martín-García et al., 2014). GABAergic interneurons have not been manipulated yet in addiction models, but the role of PV interneurons in natural reward (sucrose) learning and extinction was recently examined. Channelrhodopsin-2-mediated activation of PLC PV interneurons did not affect acquisition of sucrose reward self-administration, but accelerated extinction of reward seeking by inhibiting PL network activity (Sparta et al., 2014). Whether PLC PV activity also affects extinction of drug seeking remains a topic for future research.

By integrating input from sources such as the BLA, VTA and HPC and conveying excitatory output to the NAc, the mPFC is thought to exert control over the motor circuitry to regulate responding to drugs and drug-associated stimuli (Kalivas et al., 2005). Dorsal regions of the mPFC mainly project to the dorsolateral striatum and NAc core, whereas ventral regions predominantly target the dorsomedial striatum and NAc shell (Voorn et al., 2004). Pharmacological disconnection experiments have indeed implicated the dmPFC-NAc core and vmPFC-NAc shell pathway in drug- and cue-induced cocaine and heroin seeking (McFarland et al., 2003; LaLumiere and Kalivas, 2008; Peters et al., 2008; Bossert et al., 2012), but with this method the effects on indirect pathways cannot be ruled out. Photoinhibiton of PLC presynaptic terminals in the NAc core attenuated cocaine-primed reinstatement of cocaine seeking (Stefanik et al., 2013), confirming that a monosynaptic glutamatergic projection from PLC to NAc core has a critically role in this behavioral response. Optogenetic evidence for the involvement of the mPFC-NAc shell pathway was provided by a optic modulation of ILC terminals in NAc brain slices obtained from animals that were exposed to cocaine (Suska et al., 2013). This revealed that presynaptic input of mPFC terminals in the NAc shell was strengthened after both short- (1 day) and long-term (45 days) abstinence from non-contingent and contingent exposure to cocaine, but only after contingent exposure this strengthening significantly increased over time. The presynaptic enhancement was caused by an increase in glutamate release probability, rather than increased quantal size of glutamatergic release or the number of active release sites (Suska et al., 2013). Interestingly, cocaine exposure did not affect presynaptic transmission in the BLA-NAc shell projection (Suska et al., 2013), suggesting that input from the mPFC is favored over BLA input after cocaine administration. In an elegant study by Ma et al. (2014) it was shown that cocaine self-administration induced silent synapses in the mPFC-NAc pathway. Interestingly, silent synapses in the ILC-NAc shell pathway matured by recruiting GluA2-lacking AMPA-Rs (observed at day 45 of abstinence), whereas silent synapses in the PLC-NAc core pathway recruited GluA2-containing AMPA-Rs. α-amino-3-hydroxy-5-methyl-4-isoxazolepropionic acid receptors lacking the GluA2 subunit are calcium permeable, have greater channel conductance, exhibit faster channel deactivation kinetics and thereby contribute to rapid synaptic signaling, homeostatic synaptic scaling and specialized forms of short- and long-term plasticity (for excellent review see Isaac et al., 2007). Optogenetically evoked long-term depression (1 Hz for 10 min) reintroduced silent synapses in both pathways, but this either enhanced (ILC-NAc shell) or reduced (PLC-NAc core) subsequent cocaine seeking (Ma et al., 2014), further supporting differential roles of the dmPFC and vmPFC in this behavior.

The principal cell population in the NAc consists of GABAergic medium spiny neurons (MSNs) that can be subdivided in a D1-R and D2-R expressing population, together comprising ~90–95% of all NAc neurons (Lobo et al., 2006). Selective expression of ChR2 in each NAc MSN population showed that activation of D1-R neurons enhanced cocaine reward learning in the CPP paradigm, whereas activation of D2-R neurons had the opposite effect (Lobo et al., 2010). Photostimulation of mPFC terminals in the NAc core specifically induced ΔFosB expression in D1-R neurons, whereas in the NAc shell, ΔFosB expression was induced in both D1-R and D2-R subtypes (Lobo et al., 2013). This suggests that the distribution of mPFC terminals onto NAc neurons differs for the shell and core (Lobo et al., 2013). However, this will require validation by whole-cell recordings. The functional relevance of mPFC to NAc D1-R MSNs projections was demonstrated by Pascoli et al. (2012) who showed that low frequency (1 Hz) photostimulation of the ILC-NAc shell pathway reversed non-contingent cocaine-induced synaptic potentiation in D1-R neurons and locomotor sensitization. More recently, the same group used optogenetics to reveal the presence of GluA2-lacking AMPA-Rs in the ILC-NAc D1-R MSN projection 1 month after cocaine self-administration (Pascoli et al., 2014). Photostimulation of this pathway at 13 Hz, but not 1 Hz, reversed synaptic adaptations after cocaine self-administration and abolished cue-induced cocaine seeking. The authors speculated that a 13-Hz stimulation was required for this effect because this evokes mGluR-mediated long-term depression, an efficient mechanism to remove synaptic GluA2-lacking AMPA-Rs (Lüscher and Huber, 2010). However, this finding contradicts with the observation by Ma et al. (2014); (discussed above). Differences in circuit specificity (optogenetic modulation of projections to D1-R neurons *vs*. projections to all NAc shell MSN neurons) and in the cocaine self-administration regimen may explain the opposing effects observed in these studies.

In addition to being involved in relapse to drug seeking, the mPFC-NAc pathway has been implicated in compulsive aversion-resistant alcohol consumption. Photoinhibition of the dmPFC-NAc core projection reduced alcohol intake paired with aversive stimuli of different sensory modalities and different methods of intake (Seif et al., 2013). Alcohol intake was unaffected by photoinhibition when it was not paired with an adverse consequence, suggesting that this pathway predominates in orchestrating the aversion-resistant, compulsive aspects of alcoholism, in which intake is often accompanied by conflict or challenge (Tiffany and Conklin, 2000). However, these results contradict with the finding that photoinhibition of the PLC enhances aversion-resistant cocaine intake (Chen et al., 2013), suggesting that the PLC might differentially regulate compulsive alcohol and cocaine intake.

The involvement of the mPFC-NAc pathway in acquisition of reward and drug self-administration has also been explored with optogenetic approaches. Stuber et al. (2011) found that optical activation of the mPFC-NAc shell projection (20 Hz) did not support the acquisition of operant self-stimulation behavior (active responses triggered light pulses delivered to presynaptic mPFC terminals in the NAc), despite the fact that optical activation of the mPFC projection elicited EPSCs in the NAc. A subsequent study demonstrated that animals acquire optical self-stimulation of the mPFC-NAc shell pathway when the frequency of stimulation is increased to 30 Hz (Britt et al., 2012). Hence, the glutamatergic projection from the mPFC to NAc may only evoke spiking of MSNs and reinforce behavior with strong activation of the mPFC or when DA levels in the NAc are elevated in parallel. The exact stimulation site within the mPFC may be of critical importance to achieve this effect, considering that the ILC is thought to have a stronger projection to the NAc shell than the PLC (Voorn et al., 2004). As in the above mentioned studies ChR2 expression was not specifically targeted to the PLC or ILC, it remains to be determined whether a difference exists in the potency of both pathways to evoke spiking in NAc shell MSNs and to reinforce reward seeking behavior.

In line with traditional intervention techniques, optogenetic manipulations of the mPFC circuitry in rodent addiction models have validated the critical involvement of this region in regulating drug taking and drug seeking behavior and further support a functional segregation along the dorsal-ventral axis of the mPFC. Moreover, pathway specific modulation has provided new insights in the role of BLA-PLC and mPFC-NAc projections. In particular, optic stimulation of PLC and ILC axonal terminals in acute brain slices preparations of the NAc core and shell demonstrated cocaine-induced pathway-specific neuroadaptations that could be reversed using defined photoactivation frequencies (Pascoli et al., 2012, 2014; Ma et al., 2014). This may provide opportunities for DBS-mediated reversal of drug-induced neuroadaptations in addicts. However, as electrical stimulation affects neuronal activity in a non-selective manner, translational efficacy to DBS remains to be approached with caution and requires further studies.

## Concluding remarks

The relatively recent application of optogenetic technology to neuroscience research has deepened insight into function of various types of circuitry in the brain, and already contributed substantially to our understanding of the mPFC circuitry in health and disease conditions. Optogenetic manipulations enable causal system-level research on diverse cognitive and neuropathological behaviors in freely moving animals and allow integration of *in vivo* and *ex vivo* electrophysiological recordings, which was not feasible with traditional intervention methods. However, over decades, the extensive body of research involving lesion, pharmacological and electrophysiological methods has provided crucial knowledge on the involvement of the mPFC in diverse cognitive processes. Integration of data obtained with these traditional intervention methods and optogenetic modulations will continue to be invaluable for our understanding of mPFC circuitry and for creating computational models of mPFC function.

A major breakthrough in dissection of neuronal circuitries that has been enabled by optogenetics technology is the direct manipulation of neuronal projections within and between brain regions. With respect to the mPFC circuitry, this has led to a better understanding of intra-mPFC connectivity, the role of afferent and efferent mPFC projections in cognitive processes and mental disorders, and even to the discovery of a new GABAergic cell population with long-range projections to the NAc (Lee et al., 2014c). Moreover, due to the excellent compatibility of optogenetics and *ex vivo* brain slice physiology, differential cocaine-induced neuroadaptations in PLC and ILC projections to the NAc have been elucidated (Ma et al., 2014), demonstrating the feasibility of dissection of mPFC subregion-specific mechanisms using optogenetics.

Although great progress has been made, several factors have received little attention and in some cases require technical improvements to be properly addressed in future experiments. With respect to the GABAergic interneuron population in the mPFC, opsin expression has thus far been primarily targeted to PV interneurons, leaving the role of many other GABAergic cell-types (e.g., SOM+, calretinin+ cells, etc.) unaddressed. As transgenic mouse and rat Cre-driver lines become increasingly available, this opens new avenues to investigate the role of other mPFC subpopulations in cognitive performance and psychiatric disorders. Importantly, previous optogenetic studies have pointed to the existence of subpopulations within the GABAergic and pyramidal cell population that may only be distinguished based on their differential activity during defined behavioral states (Little and Carter, 2013; Courtin et al., 2014). For instance, PV interneurons have been linked to working memory performance (Rossi et al., 2012), expression of fear responses (Courtin et al., 2014), maintaining a proper E/I balance (Yizhar et al., 2011b; Kvitsiani et al., 2013), and synchronization of gamma oscillations (Sohal et al., 2009; Sohal, 2012). Optogenetic tagging of neurons that show increased activity during a particular behavioral task will be a crucial next step to dissect the causal involvement of these specific neuronal ensembles in expression of behavioral performance (Cruz et al., 2013). Opsin expression driven by the promoter of the immediate early gene *c-fos*, a widely used marker of neuronal activity, in hippocampal neurons that were active during fear-conditioning demonstrates that this is an attainable goal (Liu et al., 2012). Interpretation of optogenetic data is often hindered by non-specific targeting of opsins to mPFC subregions. As it becomes increasingly clear that dorsal and ventral regions of the mPFC have different and sometimes even opposing functions (Heidbreder and Groenewegen, 2003; Van den Oever et al., 2010), stereotactic delivery of opsin vectors to these defined subregions is of high relevance. Furthermore, technical advances that enable targeting of opsins to specific layers within the mPFC would be of great value given the complex layer- and subregion-defined neuronal connectivity of mPFC neurons (Groenewegen et al., 1997; Voorn et al., 2004; Hoover and Vertes, 2007).

Currently, many FDA approved pharmaceutical agents target G-protein coupled receptors in the brain (Lee et al., 2014b). Thus, improving insight in the temporal role of these receptors to specific behavioral states will be instrumental for treatment of psychiatric disorders with novel, more-selective pharmacotherapy. Design of opsins that consist of a chimera of an opsin fused to the intracellular domain of a G-protein coupled receptor (optoXR), enables interrogation of the causal involvement of G-protein coupled signaling cascades with high spatiotemporal resolution (Airan et al., 2009). Thus far, optoXRs have not been used to study the contribution of specific signaling cascades to mPFC circuitry function, but would be extremely useful for explaining the role of altered G-protein signaling observed in psychiatric diseases (Hearing et al., 2012; Luján et al., 2014). In addition, new developments in the field of chemogenetic technology (e.g., DREADD: Designer Receptors Exclusively Activated by Designer Drugs) will further contribute to the dissection of mPFC circuitry and the identification of drugable targets (Sternson and Roth, 2014).

The use of optogenetics in humans for treatment of neurological disorders has been extensively discussed (Peled, 2011; Kumar et al., 2013; Touriño et al., 2013), however, clinical application of optogenetics technology is, to our knowledge, currently not feasible. Extending optogenetic methods to species beyond rodents has only been stably, safely and efficiently applied in the rhesus macaque, a non-human primate (Han et al., 2009; Diester et al., 2011; Han et al., 2011; Cavanaugh et al., 2012; Gerits et al., 2012; Jazayeri et al., 2012). Further studies and clinical trials will be required to safely express and photostimulate opsins in the human brain. Hence, in spite of high promise for clinical treatment, at present, optogenetics should primarily be regarded as a powerful toolbox to functionally dissect neural circuits in animal models of disease-related symptoms and to discover and refine targets for pharmaceutical and DBS treatment.

## Conflict of interest statement

The authors declare that the research was conducted in the absence of any commercial or financial relationships that could be construed as a potential conflict of interest.
