# Supplementary material for: Optogenetic dissection of medial prefrontal cortex circuitry
Source: Front Syst Neurosci. 2014 Dec 9;8:230. doi: 10.3389/fnsys.2014.00230 (PMC4260491; doi:10.3389/fnsys.2014.00230)
Supplement: Supplementary file 1 [file Table_1.PDF]

Table S1: Optogenetic manipulations of mPFC circuitry in mice and rats. Constructs are indicated as specified in the corresponding article. tg = transgenic mouse/rat; AAV = Adeno-Associated virus (number indicates serotype); LV = Lenti virus; HSV = Herpes Simplex virus; PRV = Pseudorabies virus

Study	Region of opsin expression	Region of optic stimulation	Construct	Neuronal Subtype	Readout
**ANATOMY – layers and connectivity of the mPFC**					
Kvitsiani et al., 2013	dmPFC	ACC	(tg)PV::Cre<br>(tg)SOM::Cre<br>(AAV2/9)EF1a::DIO-ChR2-eYFP<br>(AAV2/9)EF1a::DIO-Arch-eYFP	PV + SOM Interneurons	*in vivo* recordings
Cruikshank et al., 2012	Thalamus	mPFC (L1)	(LV)Syn1::ChR2(H134R)-eYFP-WPRE	Non-specific	*ex vivo* recordings
	PLC	mPFC (L3)	(AAV)CaMKIIα::hChR2(H134R)-eYFP	Pyramidal neurons	*ex vivo* recordings
			(tg)MR90::Cre<br>(AAV)EF1a::DIO-hChR2(H134R)-eYFP		
Little & Carter, 2013	PLC(L2)	PLC	(AAV2/9)CAG::hChR2-mCherry	Non-specific	*ex vivo* recordings
		BLA			
Lee et al., 2014a	mPFC	mPFC	(AAV)CaMKIIα::ChR2-eYFP	Pyramidal neurons	*ex vivo* recordings
			(tg)PV::Cre<br>(tg)SOM::Cre<br>(AAV)EF1α::DIO-ChR2-eYFP	PV + SOM Interneurons	*ex vivo* recordings
Land et al., 2014	mPFC	mPFC	(tg)Drd1a::Cre<br>(AAV)EF1α::DIO-ChR2-eYFP	D1-R pyramidal neurons	*ex vivo* recordings
Little & Carter, 2012	MTN	PLC (L2)	(AAV)ChR2-Venus<br>(AAV)hChR2-mCherry	Pyramidal neurons projecting to mPFC	*ex vivo* recordings
	mPFC				
	BLA				
	vHPC				
Ji & Neugebauer, 2012	ILC	ILC	(AAV5)CaMKIIα::ChR2(H134R)-eYFP	Pyramidal neurons	*ex vivo* recordings
Lee et al., 2014b	mPFC	NAcc	(tg)Dlxi12b::Cre<br>(AAV5)EF1α::DIO-ChR2-eYFP	GABAergic neurons	*ex vivo* recordings<br>Real-time Place Preference
Hübner et al., 2014	mPFC	BLA	(AAV)CAG::hChR2(H134R)-mCherry<br>(AAV)hSyn::hChR2(H134R)-eYFP	Non-specific	*ex vivo* recordings
**COGNITION - working memory, alertness and temporal control**					
Gilmartin et al., 2013	PLC	PLC	(AAV9)CAG::ArchT-GFP	Non-specific	*in vivo* recordings<br>Fear-conditioning
Rossi et al., 2012	PLC	PLC	(tg)PV::Cre<br>(AAV5)EF1α::DIO-hChR2(H134R)-eYFP	PV interneurons	Delayed Alternation Task
Zhang et al., 2013	LC	mPFC	(tg)TH::IRES-Cre<br>(AAV5)EF1α::DIO-hChR2(H134R)-eYFP	NA neurons	*ex vivo* recordings
Carter et al., 2010	LC	LC	(tg)TH::IRES-Cre<br>(AAV5)Ef1α-eNpHR-eYFP	NA neurons	*ex vivo* recordings<br>*in vivo* recordings<br>Wakefulness
Narayanan et al., 2012	mPFC	mPFC	(tg)D1::Cre<br>(AAV)EF1α::DIO-hChR2(H134R)-eYFP	D1-R pyramidal neurons	*ex vivo* recordings<br>Fixed-interval Timing task
			(tg)D1::Cre<br>(AAV)EF1α::DIO-eNpHR 3.0-eYFP		
**COGNITION – learning, memory and extinction**					
Courtin et al., 2014	dmPFC	dmPFC	(tg)PV::IRES-Cre<br>(AAV9)FLEX-ArchT-GFP<br>(AAV5)EF1α::DIO-hChR2(H134R)-eYFP	PV Interneurons	*in vivo* recordings<br>Fear-conditioning
Cho et al., 2013	mPFC	BLA	(AAV5)CaMKIIα:: ChR2(H134R)-eYFP	Pyramidal neurons	*ex vivo* recordings<br>Fear-conditioning
		mPFC			
**COGNITION – habitual behavior**					
Smith et al., 2012	ILC	ILC	(AAV5)CaMKIIα::eNpHR3.0-eYFP	Pyramidal	*in vivo* recordings

				neurons	T-maze task
Smith & Graybiel 2013	ILC	ILC	(AAV5)CaMKIIα::eNpHR3.0-eYFP	Pyramidal neurons	*in vivo* recordings<br>T-maze task
**PSYCHIATRIC DISORDERS – depression**					
Convington et al. 2010	mPFC	mPFC	(HSV)IE4/5::ChR2-mCherry	Non-specific	Social Defeat
Kumar et al., 2013	PLC (L5)	PLC (L5)	(tg)Thy1::ChR2-eYFP	Pyramidal neurons	*in vivo* recordings<br>Social Defeat
Warden et al., 2012	mPFC	DRN<br>LHb	(AAV5)CaMKIIα::ChR2-eYFP	Pyramidal neurons	*in vivo* recordings<br>Force Swim Test
Challis et al., 2014	vmPFC	DRN	(AAV2/9)CaMKIIα::ChR2-eYFP<br>(tg) CaMKIIα ::Cre<br>(AAV2/9)EF1α::DIO-hChR2(H134R)-eYFP<br>(AAV2/9)CBA::flex-Arch-GFP	Pyramidal neurons	*ex vivo* recordings<br>Social Defeat
Vialou et al., 2014	PLC	NAc<br>BLA	(AAV)CaMKIIα::ChR2-eYFP	Pyramidal neurons	Social Defeat
Chaudhury et al., 2013	VTA	VTA (DA neurons projecting to mPFC)	(PRV)Cre<br>(AAV)DIO-ChR2-eYFP<br>(AAV)DIO-NpHR-eYFP	VTA DA neurons	*in vivo* recordings<br>Social Defeat
Friedman et al., 2014	VTA	VTA (mPFC projections)	(tg)TH::IRES-Cre<br>(AAV)DIO-ChR2-eYFP	VTA DA neurons	*ex vivo* recordings<br>Social Defeat
Gunaydin et al., 2014	VTA	mPFC	(tg)TH::Cre<br>(AAV5)EF1α::DIO-ChR2-eYFP	VTA DA neurons	*in vivo* fiber photometry<br>Social Interaction<br>Place avoidance
**PSYCHIATRIC DISORDERS – schizophrenia**					
Gee et al., 2012	mPFC	mPFC	(tg)Drd1::Cre<br>(AAV)DIO-ChR2-eYFP	D1-R pyramidal neurons	*ex vivo* recordings
			(tg)Drd2::Cre<br>(AAV)DIO-ChR2-eYFP	D2-R pyramidal neurons	
			(AAV)CaMKIIα::ChR2-eYFP	Pyramidal neurons	
Yizhar et al., 2011	mPFC	mPFC	(AAV5)CaMKIIα::SSFO-eYFP<br>(AAV5) CaMKIIα::C1V1-eYFP	Pyramidal neurons	*in vivo* recordings<br>Social Interaction<br>Fear Conditioning
		mPFC	(tg)PV::Cre<br>(AAV5)EF1α::DIO-SSFO-eYFP<br>(AAV5)EF1α::DIO-C1V1-eYFP	Interneurons	
Sohal et al., 2009	mPFC	mPFC	(tg)PV::Cre<br>(AAV5) EF1α::DIO-eNpHR-eYFP<br>(AAV5)EF1α::ChR2 -eYFP	Interneurons	*in vivo* recordings<br>*ex vivo* recordings
			(tg)Thy1::ChR2-eYFP	Pyramidal neurons	
**PSYCHIATRIC DISORDERS – addiction**					
Chen et al., 2013	PLC	PLC	(AAV)CaMKIIα::ChR2-eYFP<br>(AAV)CamKIIα::eNpHR3.0-eYFP	Pyramidal neurons	*ex vivo* recordings<br>Cocaine Seeking
Van den Oever et al., 2013	vmPFC	vmPFC	(tg)CamKIIα::Cre<br>(AAV2) EF1α::DIO- ChR2(H134R)-eYFP<br>(AAV2) EF1α::DIO-eNpHR 3.0-eYFP	Pyramidal neurons	*ex vivo* recordings<br>Cocaine Conditioned Place Preference
			(AAV2)CaMKIIα:: ChR2(H134R)-eYFP<br>(AAV2)CaMKIIα::eNpHR3.0-EYFP		
Stefanik et al., 2013	PLC	PLC	(AAV2)hSyn::eNpHR3.0-eYFP	Non-specific	Cocaine Seeking
		NAc core	(AAV2)CAG-ArchT-GFP	Non-specific	
Stefanik & Kalivas, 2013	BLA	PLC	(AAV2)CAG-ArchT-GFP	Non-specific	Cocaine Seeking
Calu et al., 2013	dmPFC	dmPFC	(pAAV)CaMKIIα::eNpHR3.0-eYFP	Pyramidal neurons	*in vivo* recordings<br>Food Seeking
Sparta et al., 2014	PLC	PLC	PV::Cre<br>(AAV)EF1α::DIO-ChR2(H134R)-eYFP	Interneurons	*ex vivo* recordings<br>*in vivo* recordings<br>Sucrose seeking

Suska et al., 2013	ILC	NAc	(AAV2)CAG::ChR2Y(h134R)-eYFP	Non-specific	*ex vivo* recordings
Martin-Garcia et al., 2014	PLC	PLC	(AAV5)CaMKIIα::hChR2(H134R)-eYFP<br>(AAV5)CaMKIIα::ArchT-eYFP	Pyramidal neurons	Cocaine Taking<br>Cocaine Seeking
Ma et al., 2014	ILC	NAc shell	(AAV2)CaMKIIα::hChR2(H134R)-venus	Pyramidal neurons	*ex vivo* recordings<br>Cocaine Seeking
	PLC	NAc core			
Pascoli et al., 2012	ILC	NAc shell	(AAV1)ChR2-eYFP	pyramidal neurons	*ex vivo* recordings<br>Locomotor sensitization
Pascoli et al., 2014	ILC	NAc	(AAV1)CAG::ChR2-eYFP	pyramidal neurons	*ex vivo* recordings<br>*in vivo* recordings<br>Cocaine Seeking
Seif et al., 2013	mPFC	NAc core	(AAV)CaMKIIα::eNpHR3.0-eYFP	Pyramidal neurons	*ex vivo* recordings<br>Alcohol Taking<br>Alcohol Seeking
Stuber et al., 2011	mPFC	NAc	(AAV5)CaMKIIα::hChR2(H134R)-eYFP	Pyramidal neurons	Optical self-stimulation
Britt et al., 2012	mPFC	NAc shell	(AAV)CaMKIIα::ChR2-eYFP<br>(AAV)CaMKIIα::NpHR-eYFP	Pyramidal neurons	*ex vivo* recordings<br>Locomoter sensitization<br>Place preference<br>Optical self-stimulation
